# Porcine Airway Organoid-Derived Well-Differentiated Epithelial Cultures as a Tool for the Characterization of Swine Influenza a Virus Strains

**DOI:** 10.3390/v16111777

**Published:** 2024-11-15

**Authors:** Nora M. Gerhards, Manouk Vrieling, Romy Dresken, Sophie Nguyen-van Oort, Luca Bordes, Jerry M. Wells, Rik L. de Swart

**Affiliations:** 1 Wageningen Bioveterinary Research, 8221 RA Lelystad, The Netherlands; nora.gerhards@wur.nl (N.M.G.); manouk.vrieling@wur.nl (M.V.); luca.bordes@wur.nl (L.B.); 2 Host-Microbe Interactomics Group, Wageningen University, 6708 WD Wageningen, The Netherlands; jerry.wells@wur.nl

**Keywords:** air–liquid interface, swine influenza virus, airway epithelial cells, Transwell cultures, pig, organoids

## Abstract

Swine influenza A viruses (IAVsw) are important causes of disease in pigs but also constitute a public health risk. IAVsw strains show remarkable differences in pathogenicity. We aimed to generate airway organoids from the porcine lower respiratory tract and use these to establish well-differentiated airway epithelial cell (WD-AEC) cultures grown at an air–liquid interface (ALI) for in vitro screening of IAVsw strain virulence. Epithelial cells were isolated from bronchus tissue of juvenile pigs, and airway organoids were cultured in an extracellular matrix in a culture medium containing human growth factors. Single-cell suspensions of these 3D organoids were seeded on Transwell filters and differentiated at ALI to form a pseudostratified epithelium containing ciliated cells, mucus-producing cells and tight junctions. Inoculation with a low dose of IAVsw in a low volume inoculum resulted in virus replication without requiring the addition of trypsin, and was quantified by the detection of viral genome loads in apical washes. Interestingly, inoculation of an H3N2 strain known to cause severe disease in pigs induced a greater reduction in trans-epithelial resistance and more damage to tight junctions than H1N2 or H1N1 strains associated with mild disease in pigs. We conclude that the porcine WD-AEC model is useful in assessing the virulence of IAVsw strains.

## 1. Introduction

Influenza A virus (IAV) infections cause disease in multiple hosts, including humans, poultry and wild birds, but are also an important cause of respiratory tract infections in pigs [1,2]. Most swine IAV (IAVsw) infections are associated with relatively mild clinical signs, but infections can also result in severe disease and increased susceptibility to secondary infections within the porcine respiratory disease complex. Importantly, IAVsw strains pose a public health risk due to their ability to reassort, which potentially leads to variants that are more transmissible and pathogenic in humans [3]. Pigs are considered a potential ‘mixing vessel’ for the exchange of IAV genetic segments, as they are susceptible to human, avian and porcine IAV strains. If a single host cell is infected by two different IAV strains, the exchange of gene segments can lead to a virus with a novel combination of gene segments (‘reassortment’), potentially resulting in viruses with new infection properties in swine, birds or humans [1,4,5]. A prominent example is the 2009 Swine flu pandemic, caused by swine H1N1 virus after multiple reassortment events with other influenza A viruses [6] that led to sustained human-to-human transmission. IAVsw is enzootic in pigs in Europe [7], and occasional transmission events to humans have been reported.

Influenza A viruses have a single-stranded negative-sense RNA genome consisting of eight segments and belong to the family *Orthomyxoviridae*. The two major transmembrane glycoproteins hemagglutinin (HA) and neuraminidase (NA) are used to classify IAVs into subtypes. IAVsw strains require proteolytic cleavage of a monobasic arginine of the HA precursor protein by trypsin-like serine proteases such as TMPRRS2 or HAT to facilitate the fusion of HA2 with the endosomal membrane [8,9]. Importantly, the porcine respiratory epithelium possesses N-acetylneuraminic acids, the receptor used by IAV, linked to galactose both via alpha2,3 as well as via alpha2,6 linkage and can thus, in addition to IAVsw, also be infected by avian and human IAVs [9].

Traditional immortalized cell lines have low trypsin-like serine protease activity, and the propagation of IAVsw in these cells requires the addition of an exogenous protease. IAVsw strains are often propagated in Madin–Darby canine kidney cells, either modified to express additional sialic acids or not [10,11]. However, replication in immortalized cells and the addition of trypsin-like proteases to the culture media may result in culture adaptations that confound viral kinetics. Therefore, primary tissue culture, primary cell culture and organoid culture have been considered as alternatives that more closely mimic the respiratory tract epithelium of the host [12]. Primary-cell-based well-differentiated airway epithelial cell (WD-AEC) cultures differentiated at an air–liquid interface (ALI) provide a reproducible and readily available *in vitro* model with endogenous protease activity and a heterogenous cell population that mimic the *in vivo* airway epithelium. However, these cells have limited *in vitro* expansion potential. Airway organoids (AOs) provide another powerful tool for modeling the airway epithelium *in vitro*. AOs can be derived from pluripotent or tissue-residing stem cells and possess self-renewing capacity. Therefore, AOs can be expanded for subsequent use in many passages and can be cryostored and retrieved, which allows more experiments per tissue donor and increased experimental reproducibility. Moreover, it was recently shown that human AOs can be differentiated at ALI into WD-AECs that can be used for *in vitro* viral infection studies [13,14,15].

Different porcine tissue or cell culture models have been developed to model respiratory virus infections. These include respiratory nasal explants [16,17], primary airway epithelial cells cultured via submersion in medium [18,19], precision-cut lung slices (PCLSs) [20], primary airway epithelial cells grown at ALI [17,21,22,23] and airway organoids [24,25,26]. Here, we describe the development of porcine lower respiratory tract AO-derived WD-AEC cultures which were employed to characterize three different endemic IAVsw strains of subtypes H1N1, H1N2 and H3N1 *in vitro*. We aimed to investigate whether differences in viral kinetics and virulence can be recapitulated *in vitro* using AO-derived WD-AEC cultures.

## 2. Materials and Methods

### 2.1. Isolation of Porcine Airway Organoids (AOs)

Porcine lungs were collected from pigs that were euthanized as healthy control animals in unrelated animal experiments (license AVD40100202010304, experiment number 2020.D-0024.002) or for the sampling of primary cells for diagnostics (license AVD4010020174311, experiment number 2017.D-0063.004). The animals were obtained from a high-health farm in The Netherlands. Primary bronchial epithelial cells were isolated as described previously [27] and cultured in basement membrane extract (Geltrex BME, Gibco, Thermo Fischer Scientific; Waltham, MA, USA) submerged in an airway organoid medium [28] containing 500 ng/mL Rspondin 1, 25 ng/mL FGF7, 100 ng/mL FGF10, 100 ng/mL Noggin (all Peprotech, Thermo Fischer Scientific; Waltham, MA, USA), 500 nM A83-01 (Tocris, Bio-Techne, Abingdon, UK), 5 µM Y-27632 (Bio-Connect, Huissen, The Netherlands), 500 µM SB202190 (Sigma-Aldrich, Merck Life Science NV, Amsterdam, The Netherlands), 1× B27 (Gibco), 1.25 mM N-Acetylcysteine (Sigma), 5 mM Nicotinamide (Sigma) in Advanced Dulbecco’s Modified Eagle Medium/Nutrient Mixture F-12 (Advanced DMEM/F12, Gibco) with 1× GlutaMax (Gibco), 10 mM HEPES (Lonza, Oss, The Netherlands), 1× antibiotic-antimycotic (Gibco) and 0.1 mg/mL primocin (InVivogen, Toulouse, France). Briefly, the main bronchi were dissected and digested overnight at 4 °C on a platform rocker in DMEM/F12 (Gibco) with 10 µg/mL DNAse (Sigma-Aldrich), 1 mg/mL protease XIV (Sigma-Aldrich), 1× antibiotic-antimycotic and 0.1 mg/mL primocin (InVivogen). Subsequently, epithelial cells were harvested by mechanical scraping and strained through a 70 µm filter (Corning, Merck Life Science NV, Amsterdam, The Netherlands). Red blood cells were lysed using Red Blood Cell lysis buffer (Roche, Merck Life Science NV, Amsterdam, The Netherlands). After washing in PBS, a small fraction of the pellet was resuspended in BME and seeded as 5 × 15 µL droplets per well onto a 24-well plate. Then, 500 µL of airway organoid medium was added per well and refreshed every 5 days. AOs were split every 2 weeks using TrypLE Express (Gibco), and AO cultures were maintained at 37 °C and 5% CO_2_.

### 2.2. Differentiation of AO on Air–Liquid Interface (ALI)

Nunc Cell Culture inserts in Nunc carrier plates (both Thermo Fisher Scientific, Waltham, MA, USA), with a 0.4 µm pore size, pore density < 0.85 × 10^8^ pores cm^−2^, 0.47 cm^2^ culture area, were coated with 33 µg/mL collagen IV and 70 µg/mL fibronectin (both Sigma-Aldrich). AOs (passage 4–6) were dissociated using TrypLE Express to obtain single-cell suspensions and subsequently seeded at a density of 0.5–1 × 10^5^ cells per Transwell filter starting at a liquid–liquid interface. The growth medium consisted of Airway Epithelial Cell Basal Medium (AECBM) prepared according to the manufacturer’s instructions (Promocell, Heidelberg, Germany), supplemented with 1× antibiotic-antimycotic and 0.1 mg/mL primocin. The medium was refreshed every five days. A tight monolayer formed over the course of approximately one week, after which the growth medium was removed from the apical side, creating an air–liquid interface (ALI). The following day, cultures were transferred to a basolateral differentiation medium containing bovine pituitary extract, l-epidermal growth factor, insulin (recombinant human), hydrocortisone, epinephrine, triiodo-l-thyronine, transferrin (recombinant human) and retinoic acid, as described previously [27]. Cells were maintained in the differentiation medium at 37 °C and 5% CO_2_, with medium exchanges every five days. Once per week, the apical side was washed with prewarmed PBS containing magnesium and calcium to remove excess mucus.

### 2.3. Isolation and Propagation of IAVsw Strains

H1N1 A/swine/Netherlands/Rhezerveen/CVI9121A/2012 (EPI_ISL_195178), H3N2 A/swine/Netherlands/Ysselsteyn/CVI8866A/2012 (EPI_ISL_195222) and H1N2 A/swine/Netherlands/Barger-Compascuum/CVI6324A/2012 (EPI_ISL_195176) were derived from naturally infected farmed pigs. Viruses were isolated at Wageningen Bioveterinary Research, Lelystad, The Netherlands, from lung tissue samples as described previously [29]. Early passage virus stocks were produced in Madin–Darby canine kidney (MDCK) cells: 100 uL virus stock was added to a 90% confluent MDCK monolayer on T150 tissue culture flasks using 0.5 ug/mL trypsin TPCK (Thermo Fisher). Upon the development of CPE, infection was terminated at −80 °C before the collection and subsequent centrifugation of the supernatant at 1000× *g* for 10 min at 4 °C. Virus titers were determined by titration and expressed in Tissue Culture Infectious Dose-50 per ml (TCID_50_/_mL_) using the formula of Reed and Muench.

### 2.4. TEER Measurements

To measure epithelial integrity, 400 µL prewarmed PBS was added to the apical side of WD-AECs. Next, electrodes connected to an EVOM3 volt/Ohm meter (World Precision Instruments. Sarasota, FL, USA) were placed into the apical PBS and basolateral medium. The obtained value was multiplied by the surface area of the Transwell filter and expressed in ohm × cm^2^.

### 2.5. Infection of WD-AECs with IAVsw

For IAVsw infection experiments, porcine WD-AECs (differentiated at air–liquid interface for at least 5 weeks) of three different donors were used, and experiments were repeated three times. In each experiment, four Transwell filters were infected with each IAVsw strain, of which one was fixed 24 h post-infection (hpi), while three were followed until 48 hpi. Two uninfected filters were fixed at 0 h for staining of the WD-AEC cultures. Before inoculation, an apical wash with PBS was performed to remove mucus. Subsequently, virus stocks were rapidly thawed in a 37 °C water bath and diluted in PBS to a virus concentration of 10^4^ or 10^5^ TCID_50_/_mL_. Then, 10 µL of diluted inoculum was added apically onto the WD-AECs, resulting in an inoculum per filter of 10^2^ or 10^3^ TCID_50_, respectively. After 1 h at 37 °C, four apical washes were performed to remove unbound inoculum, and a fifth wash was performed to collect and freeze a first sample for the assessment of virus loads. After 16 h, 24 h and 48 h, 400 µL PBS was added to the apical side, briefly incubated at room temperature (RT), harvested and stored at −70 °C until analysis. At 24 h and 48 h, TEER measurements were performed after the collection of apical washes, using a separate electrode per virus and with thorough cleaning between measurements of individual WD-AEC samples. At 48 hpi, filters were fixed in 4% paraformaldehyde and stored in PBS at 4 °C.

### 2.6. RNA Isolation and IAV qRT-PCR

RNA of apical washes stored in Molgen lysis buffer was isolated by an automated robot system (PurePrep 96) using the Molgen RNA isolation kit (OE00290096). A one-step quantitative reverse transcriptase PCR (qRT-PCR) was performed using the TaqMan^®^ Fast Virus 1-Step Master Mix (Applied Biosystems; Foster City, CA, USA), 500 mM of the forward (5′-CTTCTAACCGAGGTCGAAACGTA-3′) and reverse primers (5′-CACTGGGCACGGTGAGC-3′), 250 nM probe (5′-6FAM-TCAGGCCCCCTCAAAGCCGA-QSY-3′) and 2 µL RNA template. The RT-qPCR was performed on a LightCycler480 platform with the following cycling conditions: 50 °C for 5 min, 95 °C for 20 s, 95 °C for 5 s, followed by 58 °C for 15 s and 72 °C for 20 s for 45 cycles. To calculate viral copy numbers, gBlocks were ordered at IDT (Leuven, Belgium) covering the N-terminal 220 bp gene fragment of segment 7 matrix genes of Influenza A virus (A/swine/Indiana/A02751461/2023 (H1N2)).

### 2.7. Histology and Immunohistochemistry

For transverse histology sections, formalin-fixed filters were embedded in paraffin blocks according to general pathology principles and cut into 7 µm formalin-fixed paraffin-embedded (FFPE) sections for staining by hematoxylin and eosin, or for immunohistochemistry (IHC).

For IHC labeling of p63 and Muc5AC, antigen retrieval was performed for 15 min at 100 °C in 10 mM citate buffer at pH6 (DAKO) in a pressure cooker. For acetylated antigen, no antigen retrieval was performed. After the blocking of unspecific antigen binding for 30 min at RT with 1% normal goat serum, the primary antibodies were incubated in PBS with 1% BSA for 45 min at RT. The following antibodies were used: anti-p63 (clone 4A4, Abcam, dilution 1:100), anti-Muc5AC (clone 45M1, Abcam, dilution 1:100) and anti-acetylated tubulin (clone 6-11B-1, Thermo Fisher Scientific, dilution 1:100). Subsequently, the EnVision+System HRP (DAKO) was applied at RT for 30 min, followed by incubation with 3,3′-diaminobenzidine (DAB) for 5 min before counterstaining with Mayer’s hematoxylin and mounting with the Eukitt mounting medium (Sigma Aldrich, St. Louis, MO, USA).

### 2.8. Immunofluorescence

Paraformaldehyde-fixed filters were permeabilized using 0.25% Triton X-100 in PBS for 10 min at RT, followed by blocking in 5% BSA in PBS for 1 h at RT. Anti-ZO-1 Alexa Fluor 594 (clone ZO1-1A12, Thermo Fisher Scientific, dilution 1:50) was diluted in 1% BSA in PBS and incubated for 3 h at RT, while anti-Muc5AC Alexa Fluor 488 (clone 45M1, Thermo Fisher Scientific, dilution 1:50) and anti-acTub-555 (clone 7E5H8, Thermo Fisher Scientific, dilution 1:50) were added after 2 h to incubate for 1 h at RT. For staining of IAV NP, the HB65 anti-NP antibody was labeled to Alexa Fluor 488 using the ReadyLabel^TM^ Antibody labeling kit according to the manufacturer’s instructions (Thermo Fisher Scientific) and added to the primary antibody incubation (dilution 1:50), while anti-Muc5AC-488 was omitted. DAPI counterstaining for 10 min at RT was performed before mounting in ProLong Diamond Antifade mountant (Thermo Fisher scientific). Samples were imaged using the Leica Stellaris 5 WLL confocal laser scanning microscope.

## 3. Results

### 3.1. Establishment of Porcine AO-Derived WD-AECs

**Figure 1:**
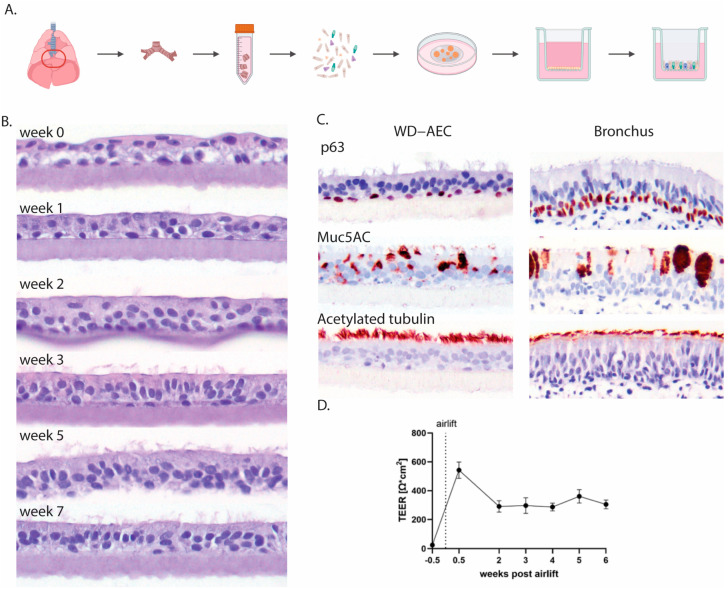
Establishment of AO-derived WD-AECs. (**A**) Schematic outline of the isolation of porcine bronchial epithelial cells from the primary bronchi which were subsequently grown as 3D organoids in an extracellular matrix before seeding in 2D on Transwell filters and culturing at air–liquid interface upon confluency. (**B**) Transverse histology sections over the course of 7 weeks showing the development of a pseudostratified ciliated respiratory epithelium. Hematoxylin and eosin stain, 40× objective. (**C**) Immunohistochemistry (IHC) staining of WD-AECs 7 weeks post-airlift and porcine bronchus epithelial cells. P63 staining to visualize basal cells, Muc5AC staining to visualize mucus (goblet cells) and acetylated tubulin staining to visualize cilia. WD-AEC resembles an *in vivo* bronchial epithelium in cellular composition and morphology, despite a reduced thickness. (**D**) Development of transepithelial electrical resistance (TEER) over the course of differentiation of WD-AECs. After an initial increase post-airlift, TEER values remained consistent. For B, C and D: Differentiation was followed for three separate donors. Representative data from one experiment are shown.

Porcine airway epithelial cells were isolated from primary bronchi, expanded in an extracellular matrix as 3D airway organoids (AOs) and subsequently seeded as single cells on Transwell filters (Figure 1A). Once the cell layers reached confluency, the apical medium was removed to generate an air–liquid interface (ALI). If sufficient tight junctions had formed, no more cell culture medium leaked through the cell layer, facilitating apical exposure to air. Subsequently, the cell layer differentiated over the course of 3–5 weeks into a pseudostratified muco-ciliated respiratory epithelium (Figure 1B) containing basal and goblet cells (Figure 1C).

**Figure 2:**
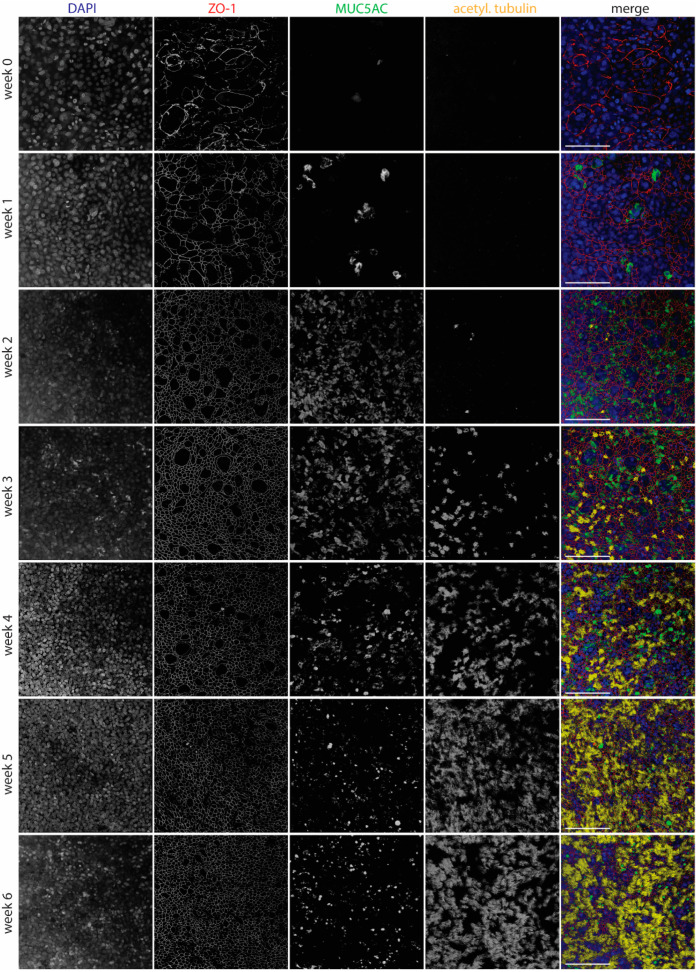
Development of WD-AECs over time. Confocal laser scanning microscopy images showing the development of tight junctions (ZO-1), goblet cells (Muc5ac) and cilia (acetylated tubulin) over the course of 6 weeks after airlifting Transwell filters. Goblet cells were most abundant at 2–4 weeks, while cilia appeared after 3 weeks. Tight junctions were detected from week 0 onwards, but only started to show a regular pattern from week 2 onwards. Merged images show nuclei in blue, tight junctions in red, goblet cells in green and cilia in yellow. Scale bar represents 100 µm. 40× objective. Representative data from one experiment are shown.

Epithelial integrity was further evaluated by measuring transepithelial electrical resistance (TEER), and it remained stable from week 2 of differentiation onwards (Figure 1D). Using laser scanning confocal microscopy, tight junctions were detected early after differentiation by staining with an antibody to ZO-1. Goblet cells, visualized by staining of MUC5AC, appeared after one week of differentiation and were most numerous at 2–4 weeks of differentiation. Cilia, stained by an anti-acetylated tubulin antibody, were visible starting from week 3 and were fully developed by week 5 (Figure 2).

### 3.2. Swine Influenza Virus Strains Replicate in Porcine WD-AECs

**Figure 3:**
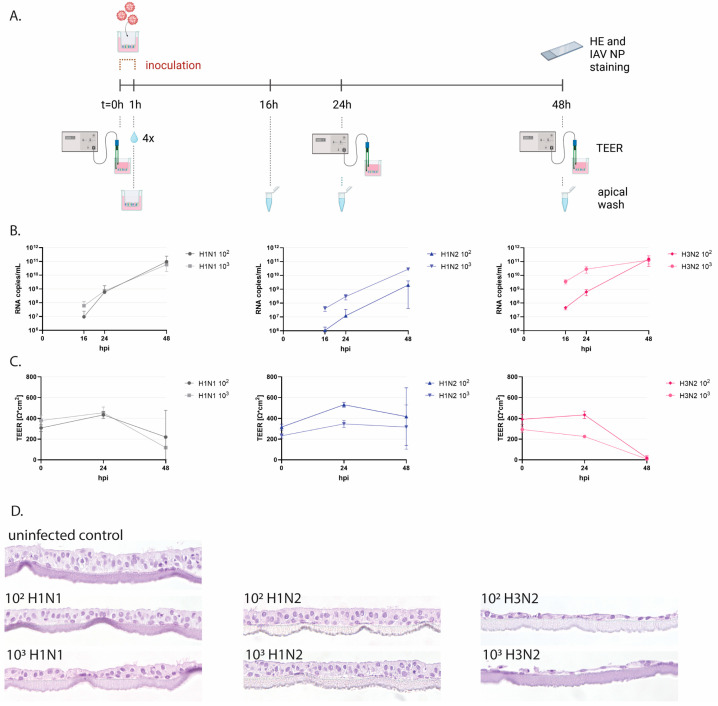
Infection of porcine WD-AECs with IAVsw. (**A**) Experimental design of IAVsw inoculation of porcine WD-AEC cultures. Three different IAVsw strains were used (H1N1, H1N2, H3N2) and applied apically at a dose of 10^2^ or 10^3^ TCID_50_ in a volume of 10 µL. After 1 h incubation, Transwell filters were washed 4 times. An apical wash was collected at 16 h, 24 h and 48 h post-inoculation, and transepithelial electrical resistance (TEER) was measured at 0 hpi, 24 hpi and 48 hpi. At 48 hpi, filters were fixed and stained by hematoxylin and eosin (HE) and against IAV nucleoprotein (NP). (**B**) Viral RNA loads in apical washes. All viruses replicated in porcine WD-AECs. For H1N2, a dose–response curve was observed over all evaluated time points, which was only observed at the earlier time points for the other two viruses. (**C**) TEER values as a measure of epithelial integrity. TEER values declined for H3N2- and H1N1-inoculated WD-AECs over 48 h but remained constant in H1N2-inoculated WD-AECs. (**D**) Transverse histology sections stained by HE at 48 h post-infection. The epithelial cell layer remained intact for H1N2-inoculated WD-AECs, while there was substantial thinning of the cell layer in H3N2-inoculated WD-AECs. For H1N1, the higher dose seemed to cause more cell loss compared to the lower dose. 40× objective. B, C and D show representative results from one out of three experiments (pig04).

To functionally evaluate porcine WD-AECs, we employed inoculations with IAVsw as an infection model (Figure 3A and Supplementary Figure S1). WD-AECs of three different porcine donors were washed with PBS to remove mucus and subsequently inoculated with 10 µL of 10^2^ or 10^3^ TCID_50_ of H1N1, H1N2 or H3N2 strains for one hour. Apical washes collected at 16, 24 or 48 hpi were analyzed by qRT-PCR. For all three virus strains, an increase in viral RNA genome loads were observed over time. For H1N2, a dose–response relationship was maintained throughout the 48 h, while for H1N1 and H3N2, a dose-dependent difference in viral RNA copies was visible only at earlier time points (Figure 3B). TEER values remained stable for H1N2-inoculated WD-AECs, while for H3N2, TEER values reached almost zero after 48 h. TEER values for H1N1-inoculated WD-AECs declined over time (Figure 3C).

### 3.3. IAVsw Strains Cause Epithelial Damage on WD-AECs

Transverse FFPE sections of WD-AECs fixed after 48 hpi demonstrated a substantial loss of cells after H3N2 inoculation, while the epithelial cell layer of H1N2-inoculated WD-AECs remained comparable to that of PBS-inoculated WD-AECs (Figure 3D and Supplementary Figure S1). For H1N1, only the 10^3^ dose appeared to cause damage to the cell layer, although the effect was much less compared to H3N2-infected cultures.

**Figure 4:**
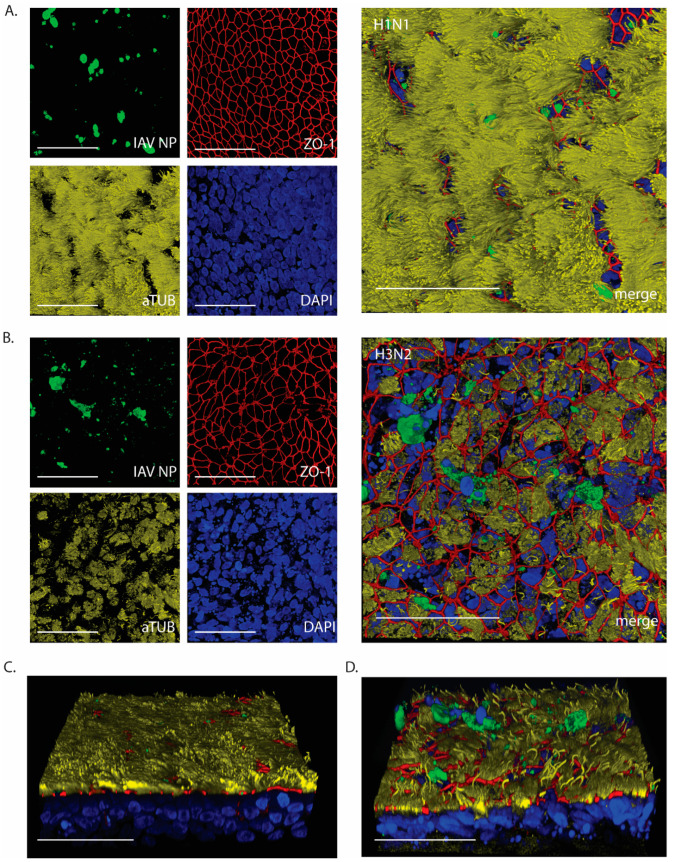
IAV NP expression after 24 h post-infection with 10^3^ TCID_50_**.** Confocal microscopy images to visualize expression of IAV NP and epithelial composition. (**A**) H1N1-inoculated WD-AECs showed NP expression (green), continuous tight junctions (red), a thick layer of cilia (yellow) and predominantly intact nuclei (blue). (**B**) H3N2-inoculated filters showed NP expression while at the same time tight junctions were compromised, cilia expression was reduced and more fragmented nuclei were visible. (**C**) Angled side-view of WD-AEC shown in (**A**). (**D**) Angled side-view of WD-AEC shown in (**B**). Note that IAV NP staining (green) is visible within or above the apical side of WD-AEC cultures. All images: Scale bar represents 50 µm. 100× objective. Representative data from one experiment are shown.

At 24 hpi, WD-AECs inoculated with 10^3^ of H1N1 or H3N2 were evaluated by confocal laser scanning microscopy (Figure 4A and Figure 4B, respectively). While the epithelium composition remained comparable to that of intact WD-AECs inoculated with H1N1 (see Figure 2), striking cytopathogenic effects were observed in H3N2-inoculated WD-AECs. Tight junctions were discontinuous, cilia were shortened and less abundant, and there was an increase in karyorrhectic debris. At the same time, IAV-infected cells were visible in both cell layers, as shown by staining against viral NP. Interestingly, IAV NP-positive cells were often located apically within or slightly above the WD-AEC layer (Figure 4C,D).

## 4. Discussion

Here, we describe the generation of porcine airway organoids from lung tissue of juvenile pigs and subsequent seeding on Transwells, leading to the generation of pseudostratified well-differentiated airway epithelial cells (WD-AEC) cultured at an air–liquid interface (ALI) to obtain an *in vitro* stem cell-derived model of the mucociliary respiratory epithelium of pigs. Subsequently, we used these WD-AEC cultures for the characterization of IAVsw strains of subtypes H1N1, H1N2 and H3N2.

The generation of porcine AOs was largely based on a protocol previously described for human airway AOs [28]. Indeed, human growth factors are sufficiently conserved to allow their use in the expansion of porcine airway epithelial stem cells [24]. However, airway organoids grown in 3D culture are not conveniently used as models for respiratory virus infection, as their cilia are usually directed inwards [28]. This is a disadvantage when mimicking the natural infection route via inhaled air, because the apical side of the respiratory epithelium is not easily accessible. This challenge can be resolved by applying protocols for culturing apical-out airway organoids, although these cultures will remain at a liquid–liquid interface [30].

We chose to prepare single-cell preparations of AOs grown in 3D, and seed these in Transwell filters to generate WD-AEC cultures grown at ALI [13,31]. By doing so, ciliated cells are facing upwards and are exposed to air, making them easily accessible for inoculation and apical washes, and the cultures resemble the bronchial epithelium found *in vivo*. A major advantage of using AO-derived WD-AECs instead of primary-cell-derived WD-AECs is the stem-cell-based renewal of organoids. Thus, cells obtained from a single donor can be expanded to high numbers, allowing the long-term use of a single donor. This increases inter-experimental comparability, allows for comparison between donors and can be used to reflect animal-to-animal variation which is observed *in vivo*.

Interestingly, during the differentiation process, we observed an increase in goblet cells at weeks 2/3 and a subsequent decrease. This coincided with an observed increased production of mucus during this time. We observed that the frequencies of goblet cells declined in subsequent weeks, when ciliated cells appeared. By staining the individual cell populations, we determined that, with our culturing conditions, porcine WD-AECs are suitable for virus infection studies after 5 weeks of differentiation. However, it should be noted that the thickness of the WD-AEC epithelium is reduced compared to the epithelium observed *in vivo*. It is unclear to what extent this affects the outcomes of the model.

Conventional cell lines require the addition of an exogenous protease for initial infection with influenza viruses with hemagglutinins containing a monobasic cleavage site. In contrast, AO-derived WD-AECs can be readily infected without the requirement of exogenous protease activity. Moreover, the high susceptibility and permissiveness of the cells was shown using a low inoculum dose of 10^2^ or 10^3^ TCID_50_, allowing room for exponential virus replication and dissemination. We chose to use two parallel doses with a tenfold difference to demonstrate that differences in *in vitro* virulence were not caused by minor dose calculation errors.

Using porcine WD-AECs, it was feasible to detect differences in viral kinetics and cytopathogenicity between the three different IAVsw strains. The strains were isolated from pigs during a field investigation in 2012, during which H3N2 viruses were the most virulent circulating strains [32]. In our experiments, H1N2 reached lower viral RNA copy numbers than H1N1 and H3N2, while no substantial differences in genome loads were observed between H3N2 and H1N1. However, when evaluating TEER values and histology, H3N2 clearly inflicted the most pronounced cytopathic effect. The high cytopathogenicity of the H3N2 strain was reproduced in three independent experiments, using tissues from three different porcine donors. Moreover, these results are in line with previously reported data using virus isolates of the same IAVsw subtypes on porcine precision-cut lung slices [20]. *In vivo*, similar cytokine responses and viral lung titers were previously observed for H3N2 and H1N1 isolates [33], although there are reports describing more severe clinical disease upon infection with H3N2 [20]. H1N2 isolates seem to cause slightly delayed immune responses compared to H1N1 and H3N2 isolates [33].

Several other studies have reported on IAVsw infections in porcine ALI models generated from primary cells. Wu et al. used a relatively high inoculum dose (approximately 10^5^ infectious units) of an H1N1 or H3N2 strain and detected viral shedding over a period of more than a week, associated with a loss of ciliated cells, but retained TEER values [21]. Krunkosky et al. used a low inoculum (250 plaque-forming units) and showed efficient virus replication in the absence of substantial drops in TEER values [17]. We consistently observed a reduction in TEER values after infection with our IAVsw H3N2 strain, coinciding with a loss of tight junction integrity and a clear loss of cells detected by histochemistry. Whether this is related to differences in the culture properties or differences between the virus strains used will need to be determined. We intend to use our AO-derived ALI model to isolate currently circulating IAVsw strains, and compare their *in vitro* and *in vivo* virulence.

This study has several limitations. We used airway organoids of three porcine donors, but in addition to donor differences, we also observed batch-to-batch differences between cultures, with substantial differences in the TEER values detected at the start of the infection. Moreover, we used virus strains that were isolated more than ten years ago. Although these had been well characterized *in vitro*, no detailed *in vivo* virulence data were available. Finally, the virus strains were isolated in MDCK cells, potentially resulting in tissue culture adaptations [19]. Future studies will be performed with primary isolates obtained in ALI cultures, thus retaining the *in vivo* properties of the virus as much as possible.

In conclusion, porcine airway organoid-derived WD-AECs offer a potential bridge between *in vivo* animal models or clinical studies and *in vitro* experiments in conventional immortalized cell lines, because of their similarity to the *in vivo* respiratory epithelium. WD-AECs therefore offer a valuable tool for characterizing new virus variants, as well as for isolating viruses from field samples. Moreover, *in vitro* studies to evaluate therapeutics, e.g., antivirals, can be performed, as well as studies to evaluate the host range of viruses when comparing WD-AECs from several animal species. The next step is to combine these WD-AEC cultures with immune cells, to recapitulate the complex interactions between the respiratory epithelium and the immune system.

## Data Availability

All data will be made available upon reasonable request.

## Supplementary Materials

The following supporting information can be downloaded at https://www.mdpi.com/article/10.3390/v16111777/s1, Figure S1: Replicates of IAVsw infection experiments on porcine WD-AECs of two different donors.

## References

[1] Castrucci M.R., Donatelli I., Sidoli L., Barigazzi G., Kawaoka Y., Webster R.G. (1993). Genetic reassortment between avian and human influenza A viruses in Italian pigs. Virology.

[2] Ma W. (2020). Swine influenza virus: Current status and challenge. Virus Res..

[3] Anderson T.K., Chang J., Arendsee Z.W., Venkatesh D., Souza C.K., Kimble J.B., Lewis N.S., Davis C.T., Vincent A.L. (2021). Swine Influenza A Viruses and the Tangled Relationship with Humans. Cold Spring Harb. Perspect. Med..

[4] Cogdale J., Kele B., Myers R., Harvey R., Lofts A., Mikaiel T., Hoschler K., Banyard A.C., James J., Mollett B.C. (2024). A case of swine influenza A(H1N2)v in England, November 2023. Euro Surveill..

[5] Hennig C., Graaf A., Petric P.P., Graf L., Schwemmle M., Beer M., Harder T. (2022). Are pigs overestimated as a source of zoonotic influenza viruses?. Porc. Health Manag..

[6] Novel Swine-Origin Influenza A.V.I.T., Dawood F.S., Jain S., Finelli L., Shaw M.W., Lindstrom S., Garten R.J., Gubareva L.V., Xu X., Bridges C.B. (2009). Emergence of a novel swine-origin influenza A (H1N1) virus in humans. N. Engl. J. Med..

[7] Simon G., Larsen L.E., Durrwald R., Foni E., Harder T., Van Reeth K., Markowska-Daniel I., Reid S.M., Dan A., Maldonado J. (2014). European surveillance network for influenza in pigs: Surveillance programs, diagnostic tools and Swine influenza virus subtypes identified in 14 European countries from 2010 to 2013. PLoS ONE.

[8] Bottcher E., Matrosovich T., Beyerle M., Klenk H.D., Garten W., Matrosovich M. (2006). Proteolytic activation of influenza viruses by serine proteases TMPRSS2 and HAT from human airway epithelium. J. Virol..

[9] Peitsch C., Klenk H.D., Garten W., Bottcher-Friebertshauser E. (2014). Activation of influenza A viruses by host proteases from swine airway epithelium. J. Virol..

[10] Harada Y., Takahashi H., Trusheim H., Roth B., Mizuta K., Hirata-Saito A., Ogane T., Odagiri T., Tashiro M., Yamamoto N. (2020). Comparison of suspension MDCK cells, adherent MDCK cells, and LLC-MK2 cells for selective isolation of influenza viruses to be used as vaccine seeds. Influenza Other Respir. Viruses.

[11] Suderman M., Moniwa M., Alkie T.N., Ojkic D., Broes A., Pople N., Berhane Y. (2021). Comparative Susceptibility of Madin-Darby Canine Kidney (MDCK) Derived Cell Lines for Isolation of Swine Origin Influenza A Viruses from Different Clinical Specimens. Viruses.

[12] Belser J.A. (2018). Cell culture keeps pace with influenza virus. Lancet Respir. Med..

[13] Lamers M.M., Mykytyn A.Z., Breugem T.I., Wang Y., Wu D.C., Riesebosch S., van den Doel P.B., Schipper D., Bestebroer T., Wu N.C. (2021). Human airway cells prevent SARS-CoV-2 multibasic cleavage site cell culture adaptation. Elife.

[14] Rijsbergen L.C., van Dijk L.L.A., Engel M.F.M., de Vries R.D., de Swart R.L. (2021). In Vitro Modelling of Respiratory Virus Infections in Human Airway Epithelial Cells—A Systematic Review. Front. Immunol..

[15] Bauer L., Rijsbergen L.C., Leijten L., Benavides F.F., Noack D., Lamers M.M., Haagmans B.L., de Vries R.D., de Swart R.L., van Riel D. (2023). The pro-inflammatory response to influenza A virus infection is fueled by endothelial cells. Life Sci. Alliance.

[16] Glorieux S., Van den Broeck W., van der Meulen K.M., Van Reeth K., Favoreel H.W., Nauwynck H.J. (2007). In vitro culture of porcine respiratory nasal mucosa explants for studying the interaction of porcine viruses with the respiratory tract. J. Virol. Methods.

[17] Krunkosky M., Krunkosky T.M., Meliopoulos V., Kyriakis C.S., Schultz-Cherry S., Tompkins S.M. (2024). Establishment of Swine Primary Nasal, Tracheal, and Bronchial Epithelial Cell Culture Models for the Study of Influenza Virus Infection. J. Virol. Methods.

[18] Sreenivasan C.C., Thomas M., Antony L., Wormstadt T., Hildreth M.B., Wang D., Hause B., Francis D.H., Li F., Kaushik R.S. (2019). Development and characterization of swine primary respiratory epithelial cells and their susceptibility to infection by four influenza virus types. Virology.

[19] Meliopoulos V., Cherry S., Wohlgemuth N., Honce R., Barnard K., Gauger P., Davis T., Shult P., Parrish C., Schultz-Cherry S. (2020). Primary Swine Respiratory Epithelial Cell Lines for the Efficient Isolation and Propagation of Influenza A Viruses. J. Virol..

[20] Meng F., Punyadarsaniya D., Uhlenbruck S., Hennig-Pauka I., Schwegmann-Wessels C., Ren X., Durrwald R., Herrler G. (2013). Replication characteristics of swine influenza viruses in precision-cut lung slices reflect the virulence properties of the viruses. Vet. Res..

[21] Wu N.H., Yang W., Beineke A., Dijkman R., Matrosovich M., Baumgartner W., Thiel V., Valentin-Weigand P., Meng F., Herrler G. (2016). The differentiated airway epithelium infected by influenza viruses maintains the barrier function despite a dramatic loss of ciliated cells. Sci. Rep..

[22] Wang H., He L., Liu B., Feng Y., Zhou H., Zhang Z., Wu Y., Wang J., Gan Y., Yuan T. (2018). Establishment and comparison of air-liquid interface culture systems for primary and immortalized swine tracheal epithelial cells. BMC Cell Biol..

[23] Peng J.Y., Shin D.L., Li G., Wu N.H., Herrler G. (2021). Time-dependent viral interference between influenza virus and coronavirus in the infection of differentiated porcine airway epithelial cells. Virulence.

[24] Jiang C., Li L., Xue M., Zhao L., Liu X., Wang W., Feng L., Liu P. (2022). Long-Term Expanding Porcine Airway Organoids Provide Insights into the Pathogenesis and Innate Immunity of Porcine Respiratory Coronavirus Infection. J. Virol..

[25] Bonillo-Lopez L., Carmona-Vicente N., Tarrés-Freixas F., Kochanowski K., Martínez J., Perez M., Sibila M., Correa-Fiz F., Aragon V. (2024). Porcine Nasal Organoids as a model to study the interactions between the swine nasal microbiota and the host. bioRxiv.

[26] Davila K.M.S., Nelli R.K., Mora-Diaz J.C., Sang Y., Miller L.C., Gimenez-Lirola L.G. (2024). Transcriptome Analysis in Air-Liquid Interface Porcine Respiratory Epithelial Cell Cultures Reveals That the Betacoronavirus Porcine Encephalomyelitis Hemagglutinating Virus Induces a Robust Interferon Response to Infection. Viruses.

[27] Bordes L., Gerhards N.M., Peters S., van Oort S., Roose M., Dresken R., Venema S., Vrieling M., Engelsma M., van der Poel W.H.M. (2024). H5N1 clade 2.3.4.4b avian influenza viruses replicate in differentiated bovine airway epithelial cells cultured at air-liquid interface. J. Gen. Virol..

[28] Sachs N., Papaspyropoulos A., Zomer-van Ommen D.D., Heo I., Bottinger L., Klay D., Weeber F., Huelsz-Prince G., Iakobachvili N., Amatngalim G.D. (2019). Long-term expanding human airway organoids for disease modeling. EMBO J..

[29] Heinen P.P., de Boer-Luijtze E.A., Bianchi A.T.J. (2001). Respiratory and systemic humoral and cellular immune responses of pigs to a heterosubtypic influenza A virus infection. J. Gen. Virol..

[30] Liu C., Diong X., Liu P., Lin X. (2024). Advances in porcine respiratory and intestinal organoids: Status and potential application for virus infections. One Health Adv..

[31] Rijsbergen L.C., Lamers M.M., Comvalius A.D., Koutstaal R.W., Schipper D., Duprex W.P., Haagmans B.L., de Vries R.D., de Swart R.L. (2021). Human Respiratory Syncytial Virus Subgroup A and B Infections in Nasal, Bronchial, Small-Airway, and Organoid-Derived Respiratory Cultures. mSphere.

[32] Stockhofe N. (2020). Personal communication.

[33] Crisci E., Mussa T., Fraile L., Montoya M. (2013). Review: Influenza virus in pigs. Mol. Immunol..

