# Supplementary material for: Porcine Airway Organoid-Derived Well-Differentiated Epithelial Cultures as a Tool for the Characterization of Swine Influenza a Virus Strains
Source: Viruses. 2024 Nov 15;16(11):1777. doi: 10.3390/v16111777 (PMC11598950; doi:10.3390/v16111777)
Supplement: Supplementary file 1 [file viruses-16-01777-s001.zip › viruses-3272282-supplementary.pdf]

Supplemental material

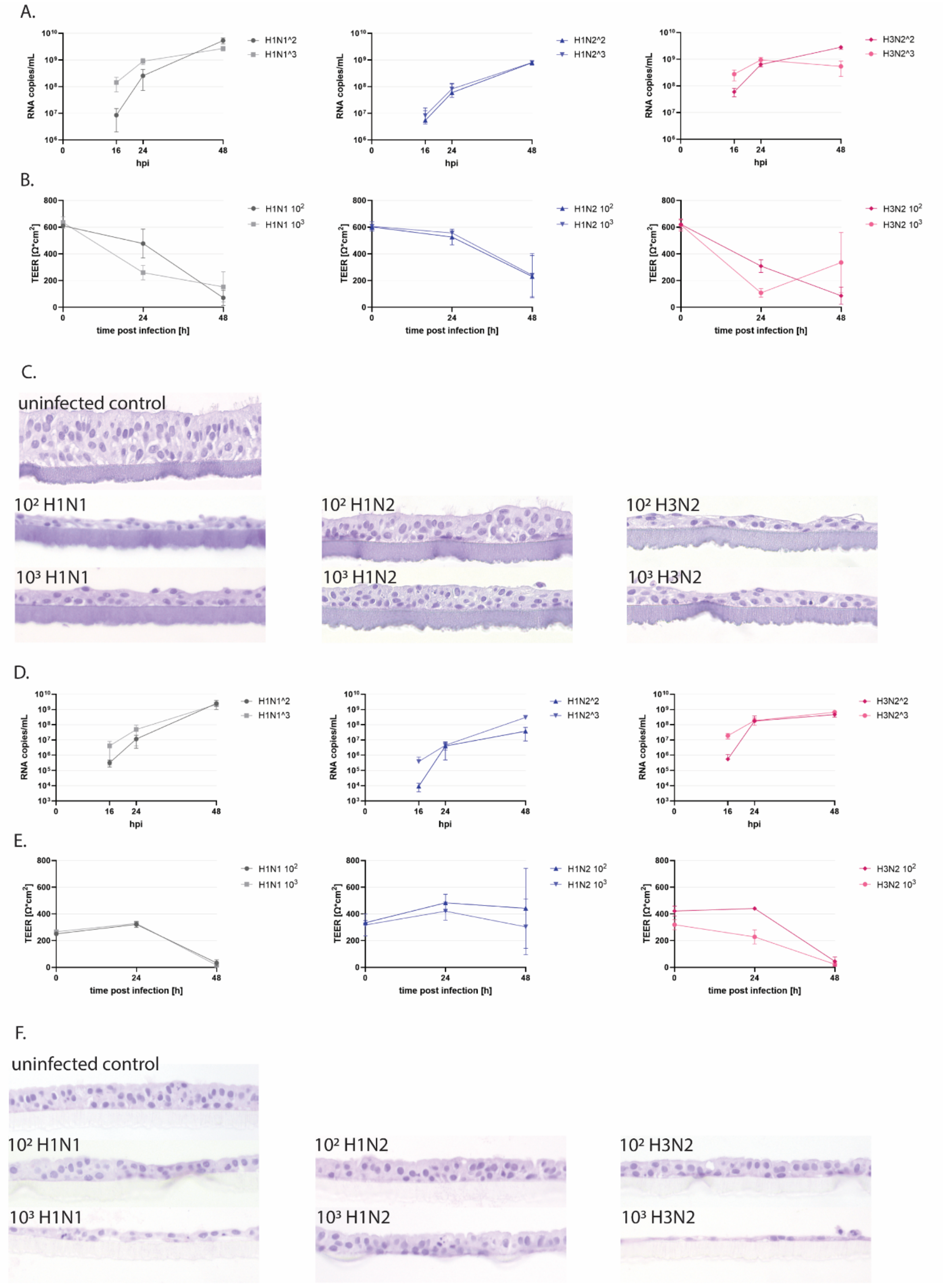

Figure S1. Replicates of IAVsw infection experiments on porcine WD-AECs of two different donors. (A), (B) and (C) show data of pig01 and (D), (E) and (F) show data of pig05. (A) and (D) Viral RNA loads in apical washes. (B) and (E) TEER values as a measure of epithelial integrity. (C) and (F) Transverse histology sections stained by HE at 48h post infection.
